# Optimization of brain PET imaging for a multicentre trial: the French CATI experience

**DOI:** 10.1186/s40658-016-0141-8

**Published:** 2016-04-05

**Authors:** Marie-Odile Habert, Sullivan Marie, Hugo Bertin, Moana Reynal, Jean-Baptiste Martini, Mamadou Diallo, Aurélie Kas, the CATI Nuclear Medicine network, Régine Trébossen

**Affiliations:** Sorbonne Universités, UPMC Univ Paris 06, CNRS, INSERM, Laboratoire d’Imagerie Biomédicale, F-75013 Paris, France; Centre pour l’Acquisition et le Traitement des Images (www.cati-neuroimaging.com), Paris, France; AP-HP, Hôpital Pitié-Salpêtrière, Département de Médecine Nucléaire, F-75013 Paris, France; CEA-Service Hospitalier Frédéric Joliot, F-91401 Orsay, France; Faculté de Médecine – Laboratoire d’Imagerie Biomédicale, 91 Boulevard de l’Hôpital, F-75013 Paris, France

**Keywords:** Neurology, PET/CT, Quality assurance, Standardization, Multicentre trials

## Abstract

**Background:**

CATI is a French initiative launched in 2010 to handle the neuroimaging of a large cohort of subjects recruited for an Alzheimer’s research program called MEMENTO. This paper presents our test protocol and results obtained for the 22 PET centres (overall 13 different scanners) involved in the MEMENTO cohort. We determined acquisition parameters using phantom experiments prior to patient studies, with the aim of optimizing PET quantitative values to the highest possible per site, while reducing, if possible, variability across centres.

**Methods:**

Jaszczak’s and 3D-Hoffman’s phantom measurements were used to assess image spatial resolution (ISR), recovery coefficients (RC) in hot and cold spheres, and signal-to-noise ratio (SNR). For each centre, the optimal reconstruction parameters were chosen as those maximizing ISR and RC without a noticeable decrease in SNR. Point-spread-function (PSF) modelling reconstructions were discarded. The three figures of merit extracted from the images reconstructed with optimized parameters and routine schemes were compared, as were volumes of interest ratios extracted from Hoffman acquisitions. The net effect of the 3D-OSEM reconstruction parameter optimization was investigated on a subset of 18 scanners without PSF modelling reconstruction.

**Results:**

Compared to the routine parameters of the 22 PET centres, average RC in the two smallest hot and cold spheres and average ISR remained stable or were improved with the optimized reconstruction, at the expense of slight SNR degradation, while the dispersion of values was reduced.

For the subset of scanners without PSF modelling, the mean RC of the smallest hot sphere obtained with the optimized reconstruction was significantly higher than with routine reconstruction. The putamen and caudate-to-white matter ratios measured on 3D-Hoffman acquisitions of all centres were also significantly improved by the optimization, while the variance was reduced.

**Conclusions:**

This study provides guidelines for optimizing quantitative results for multicentric PET neuroimaging trials.

## Background

CATI is a French platform funded in 2010 with the aim of supporting multicentre clinical trials involving neuroimaging (cati-neuroimaging.com). The main project devoted to CATI was to handle the multimodality imaging aspect of a large cohort of subjects included in a research program on Alzheimer’s disease (AD). This program, called MEMENTO, is a longitudinal study aiming at better understanding the natural history of Alzheimer’s disease. It has enrolled 2300 subjects with either light cognitive deficits or cognitive complaints that will be followed up for at least 5 years (http://clinicaltrials.gov/show/NCT01926249).

The imaging aspect of the MEMENTO study includes the acquisition of PET cerebral glucose metabolism and amyloid distribution, with MRI studies on anatomy, diffusion, and resting state. In this paper, we focus on the optimization of PET quantitative values to the highest possible per site, while reducing, if possible, the variability across centres for the MEMENTO cohort. Phantom data were acquired during a technical visit to the PET centres, prior to patients’ participation in the trial. The same phantom data were also acquired during follow-up visits after 18 months. The present study only addresses the data acquired during the site technical set-up.

Several large cohort studies on AD have been launched since the early 2000s. Among them, ADNI-1 was a forward-thinking, imaging-based multicentre clinical trial that involved 50 centres in North America, with the aim of identifying biomarkers of AD. For the PET imaging carried out in this study, the ADNI PET core determined reconstruction parameters for each scanner model and 3D-Hoffman phantoms were acquired with a standard protocol. However, discrepancies between the image characteristics from different PET centres remained high and were accounted for by degrading the spatial resolution to the lowest value among centres [1].

Other multicentre studies specifically focusing on AD have been conducted [2, 3]. In contrast to the ADNI trial, no phantom acquisition was performed prior to the study, and no post-filtering was applied to the data. Differences in spatially normalized FDG-PET scans obtained with scanners of different resolutions were minimized by the following measures: (i) restricting the analysis to voxels with intensity 80 % greater than the whole-brain mean, and (ii) excluding voxels from the uppermost 10 slices (i.e., from the top 22.5 mm of the brain) and from the lowermost 5 slices, where significant inter-scanner effects due to different fields of view have been reported [3].

The most advanced domain for PET image acquisition harmonization across multiple centres is oncological imaging. Guidelines for PET centres involved in clinical trials have been written by a group of experts, under the umbrella of the European society of Nuclear Medicine [4, 5], or the American College of Radiology Imaging Network [6]. These guidelines are based on quality checks with phantoms reproducing the imaging conditions encountered in the abdomen and the thorax in glucose metabolism studies. In addition to these tomography quality checks, to ensure the highest reproducibility of the measures extracted from the image for patient follow-up, disease evolution, and recovery after treatments, these groups have formulated recommendations for data analysis. Overall, these recommendations enable the cross-comparison of measurements from different centres. According to our knowledge, the latest guidelines for FDG-PET brain imaging were published in 2009 [7] and do not meet our objectives.

This paper presents our test protocol and the results obtained for the 22 PET centres involved in the MEMENTO cohort. French PET centres were equipped with systems set up between 2003 and 2012. No brain-dedicated high-resolution system or 2D tomography systems were included. Therefore, we chose to optimize acquisition and reconstruction parameters using phantom experiments prior to patient studies, with the aim of optimizing PET quantitative values to the highest possible per site, while reducing, if possible, the variability across centres. We also checked the impact of this harmonization on signal-to-noise ratios, as well as the exclusion of reconstruction with point-spread-function (PSF) modelling.

## Methods

### Phantom studies

Two phantom studies were acquired for the qualification process of the 22 centres, the Jaszczak phantom and the Hoffman 3D brain phantom. Standardized uptake value (SUV) measurements were also checked to assess the cross-calibration between the PET scanner and the dose calibration system.

The Deluxe Jaszczak phantom (model ECT/DLX/P) is a Plexiglas cylinder partially composed of cold rods (diameters 4.8 to 12.7 mm) and partially of six inserted hollow spheres. The main cylinder of 6060 mL was filled with a 5 kBq mL^−1^ FDG solution. Four spheres (internal diameters 7.86, 12.43, 15.43, and 24.82 mm) were filled with a 15 kBq mL^−1^ FDG solution. The remaining two spheres (internal diameters 9.89 and 31.27 mm) were filled with cold water.

The Hoffman 3D brain phantom (model BR/3D/P) was filled with a 37-to-55 kBq mL^−1^ FDG solution, taking great care to avoid the presence of bubbles.

A dynamic acquisition of 3 × 5 min was performed for each phantom. CT acquisition parameters were set such that the effective dose would be low (≤0.3 mSv), in accordance with the recommendations from the European Association of Nuclear Medicine [7].

The Hoffman phantom was positioned on a head holder when available, with the Deluxe Jaszczak phantom (20–30 kBq mL^−1^ FDG solution) below it for the simulation of a diffusion medium, as in subjects. Different reconstruction parameters were tested with varying reconstruction algorithms, number of iterations and subsets, reconstruction diameters, matrix sizes, or filters, always including those routinely used in brain studies. For the first 5 pilot centres, up to 10 sets of reconstructions were tested. Attenuation (derived from a CT scan) and scatter were corrected using standard software supplied by the scanner manufacturers.

Acquisition from a uniform cylinder filled with 5 kBq mL^−1^ FDG solution was also performed to verify cross-calibration.

The same team of two technologists certified for radiation protection prepared all the phantoms. The CATI PET project manager supervised the whole procedure and the setting of reconstruction parameters.

### Analysis of the Deluxe Jaszczak phantom studies

#### Jaszczak phantom studies

The different image reconstruction schemes were compared based on recovery coefficients (RC) computed for each sphere and spatial resolution estimated from the cold rods. RC were calculated from measurements in volumes of interest (VOIs) defined on each sphere and on the background. In-house software was developed for this purpose. It includes the following steps (all displayed for quality check):

1. For all sets of reconstructed PET volumes, the computation of the spheres’ centre of gravity, based on an automatic segmentation of the spheres.
2. The computation of the spheres’ centre of gravity based on the automatic segmentation of the spheres on CT scan volume, using the Hough algorithm [8], optimized with the incorporation of the spheres’ specifications.
3. VOIs were defined using both the CT spheres’ centre of gravity (determined in step 2) and the known sphere diameters, corrected if necessary for a mismatch between PET and CT spheres, then eroded to exclude the sphere wall. The background activity concentration was measured in VOIs obtained after the rotation of the VOIs drawn on the spheres.
4. The measurement of the mean activity in each sphere and background (BG), and calculation of RC (RC_H_ and RC_C_, respectively, for hot and cold spheres):

$$ {\mathrm{RC}}_{\mathrm{H}} = \frac{\raisebox{1ex}{$\left({M}_{\mathrm{S}} - {M}_{\mathrm{BG}}\right)$}\!\left/ \!\raisebox{-1ex}{$\left({M}_{\mathrm{S}} + {M}_{\mathrm{BG}}\right)$}\right.}{\raisebox{1ex}{$\left({A}_{\mathrm{S}} - {A}_{\mathrm{BG}}\right)$}\!\left/ \!\raisebox{-1ex}{$\left({A}_{\mathrm{S}} + {A}_{\mathrm{BG}}\right)$}\right.}\kern2em {\mathrm{RC}}_{\mathrm{C}} = \frac{\raisebox{1ex}{$\left({M}_{\mathrm{BG}} - {M}_{\mathrm{S}}\right)$}\!\left/ \!\raisebox{-1ex}{$\left({M}_{\mathrm{S}} + {M}_{\mathrm{BG}}\right)$}\right.}{\raisebox{1ex}{$\left(\ {A}_{\mathrm{BG}} - {A}_{\mathrm{S}}\right)$}\!\left/ \!\raisebox{-1ex}{$\left({A}_{\mathrm{S}} + {A}_{\mathrm{BG}}\right)$}\right.}, $$

where *M*_S_ is the activity measured in the sphere, *M*_BG_ is the activity measured in the background, *A*_S_ is the actual activity injected in the sphere, and *A*_BG_ is the actual activity injected in the background.

5) Signal-to-noise ratio was calculated as follows:

$$ \mathrm{S}\mathrm{N}\mathrm{R} = \frac{M_{\mathrm{S}\_12.43}-{M}_{\mathrm{BG}}}{{\mathrm{S}\mathrm{D}}_{\mathrm{BG}}}, $$

where *M*_S_12.43_ is the mean activity measured in the hot sphere of 12.43 mm, *M*_BG_ is the mean activity measured in the background, and SD_BG_ is the standard deviation in the background.

**Fig. 1:**
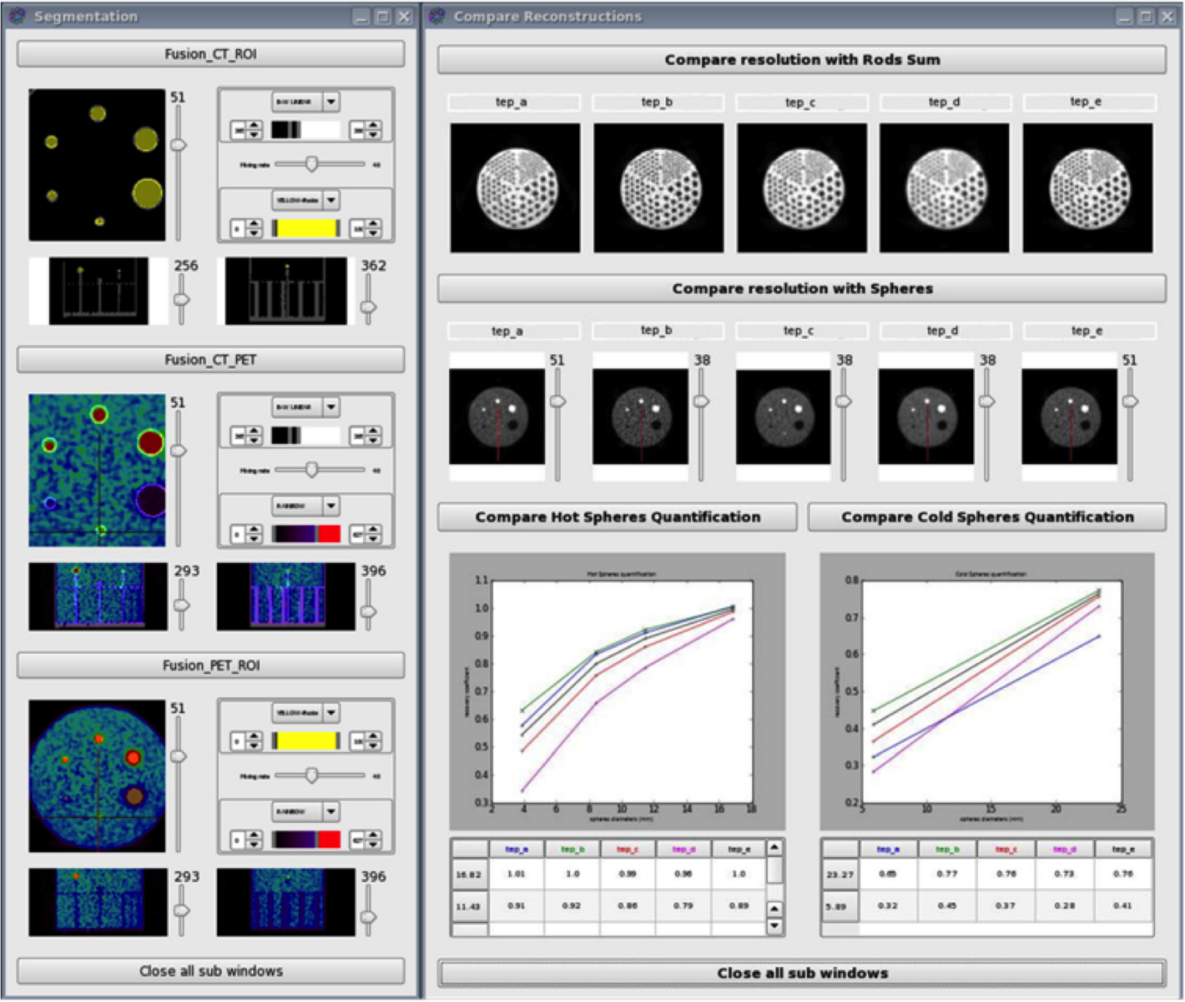
In-house software interface showing the different steps allowing the building of volumes of interest in Jaszczak phantom spheres on the *left*, and the results from the different reconstruction parameters (named *a*, *b*, *c*, *d*, and *e*) tested on the *right*

6) Display of the cold rod images and spheres for visual assessment of noise and spatial resolution, and of tables and graphics for all obtained RC according to the different reconstruction parameters (Fig. 1).

We also computed the image spatial resolution (ISR) using a method developed by Prieto et al. [9] to estimate the full width at half maximum (FWHM) of each PET scanner from the three largest hot spheres. The process can be summarized in three main steps:

1. Calculation of a ratio *R*_mes_ as follows:

$$ {R}_{\mathrm{mes}}=\frac{{\mathrm{Max}}_{\mathrm{S}}}{7{A}_{\mathrm{S}}} $$

where Max_S_ is the maximum activity measured in the sphere and *A*_S_ is the actual activity injected in the hot spheres. Each *R*_mes_ was normalized by the corresponding value of the largest sphere in order to limit the bias introduced by attenuation and scatter corrections.

2) Computation of a mask of the three spheres at the same position in the scanner field of view as during the acquisition; this mask was then convolved with a three-dimensional isotropic Gaussian function with different FWHM values ranging from 4 to 10 mm and a step of 0.1 mm. A theoretical ratio *R*_the_ was then calculated for each of the three spheres as follows:

$$ {R}_{\mathrm{the}}=\frac{{\mathrm{Max}}_{\mathrm{S}}}{A_{\mathrm{S}}} $$

where Max_S_ is the maximum value measured in the sphere after convolution and *A*_S_ is the actual value.

3) The optimal ISR value was selected as the FWHM value that best fitted the experimental values, according to minimization of the normalized sum of squared differences between observed *R*_mes_ and simulated *R*_*the*_ over the three spheres.

### Analysis of the Hoffman 3D brain phantom studies

**Fig. 2:**
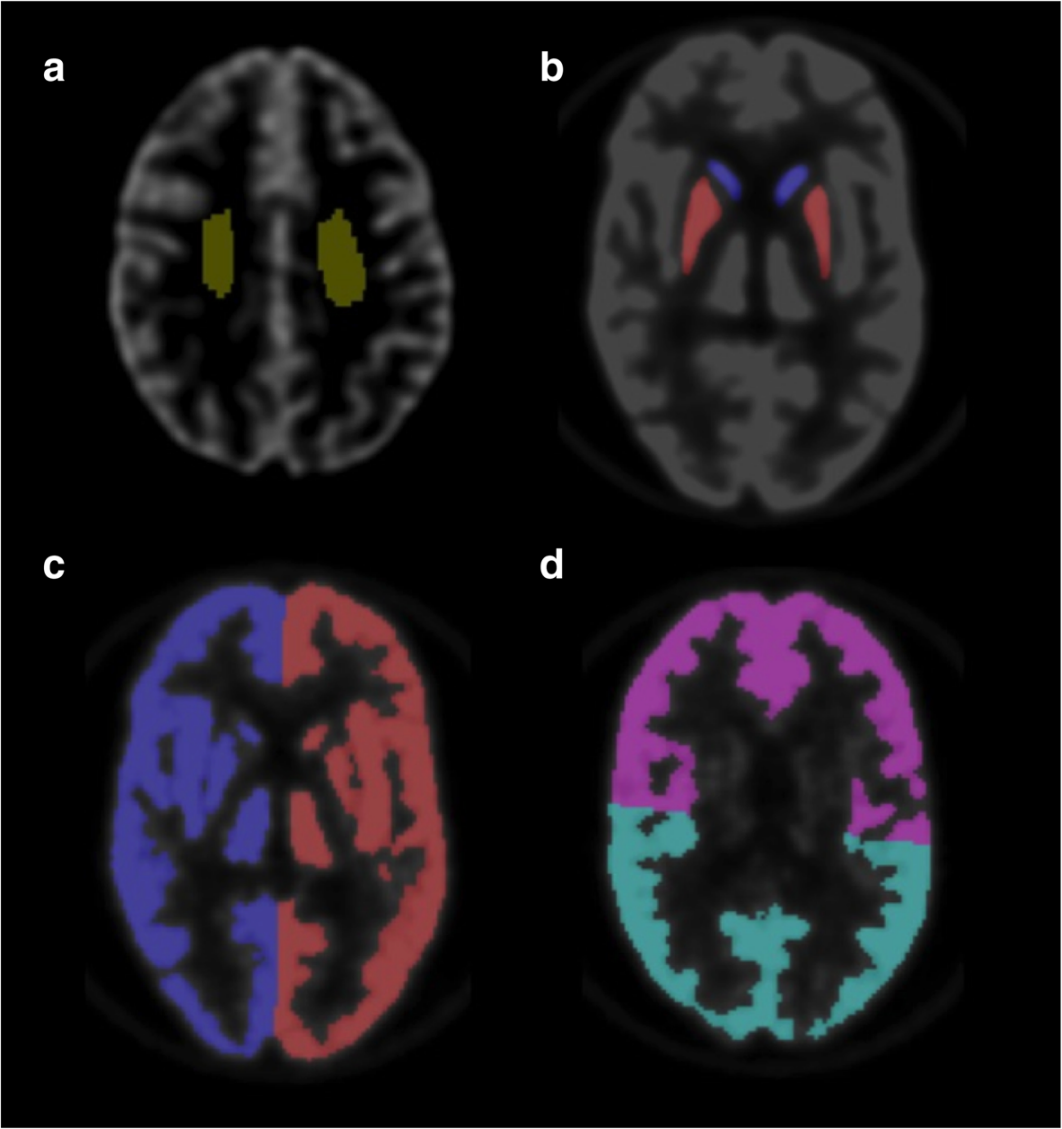
Volumes of interest used for analysis of the Hoffman 3D brain phantom acquisitions. **a** White matter. **b** Caudate (*blue*) and putamen (*red*). **c** Right and left cortex. **d** Anterior and posterior cortex

All reconstructed volumes were co-registered to the highest quality CT scan of the phantom (acquired at centre no. 16) using SPM8. The grey matter ribbon was obtained by thresholding CT image intensity at 60 % of the maximum value. The CT scan was also manually segmented in volumes of interest (Fig. 2) in order to compute right-to-left (R/L) and anterior-to-posterior (L/P) cortical ratios, as well as caudate-to-white matter (C/WM), putamen-to-white matter (P/WM), and grey-to-white matter (GM/WM) ratios from PET images. The upper and lower slices were discarded for the analyses.

### Image reconstruction

First, the three frames were summed. We then systematically compared phantom measurements obtained from images reconstructed with the algorithm selected for diagnostic purposes by each centre with the images reconstructed using the parameters optimized by CATI. Reconstruction algorithms incorporating the modelling of the spatial resolution of the tomographs were used for diagnostic purposes in four (4/22) centres. These algorithms were discarded by CATI to avoid possible additional centre effects, and 3D-OSEM statistical image reconstruction algorithms, or FORE 2D-OSEM when the 3D-OSEM was not available, were selected.

The optimization was based on three figures of merit extracted from measurements on a Jaszczak phantom, the RC of spheres of various diameters, the signal-to-background-noise ratio (SNR) and the ISR. The image reconstruction parameters were chosen at each site as a compromise between maximized RC values, ISR, and SNR. Volumes of interest ratios extracted from Hoffman acquisitions were also compared to confirm the choice of reconstruction parameters for each site. Our approach can be described as follows:

1. Reconstruction matrix was set to obtain pixel spacing inferior to 3 mm
2. The number of total iterations multiplied by the number of subsets was chosen such as the product iterations × subsets was superior to 50, and optimized with post-reconstruction smoothing
3. RC and ISF obtained with different combinations of reconstruction parameters were compared first: the parameters giving highest values with acceptable SNR were chosen.
4. Hoffmann images were then quantitatively and visually checked for the best compromise between spatial resolution and noise.
5. The optimized quantitative values for both phantoms obtained at each centre were finally compared to the routine values.

The optimized parameters chosen according to the model of scanner are presented in Table 1.

**Table 1:** Optimized parameters chosen according to the model of the scanner

GE models	Number	Installation year	Slice thickness (mm)	Recons. method	Number of iterations (iterations × subsets)	Post-reconstruction filter type and FWHM if relevant (mm)	Matrix	Pixel spacing (mm × mm)	Others
Discovery 690	3	2009/2010/2011	3.27	VPHD	10 × 36	Gaussian 3.5	256 × 256	1.17 × 1.17	Z-filter: standard
Discovery RX	2	2009	3.27	FORE + OSEM 2D	7 × 35	Gaussian 3.5	128 × 128	2.34 × 2.34	Loop filter: 2.34 mm Z-filter: standard
Discovery DST - E	1	2004	3.27	FORE + OSEM 2D	5 × 35	Gaussian 2.57	128 × 128	2.34 × 2.34	Loop filter: 2.34 mm Z-filter: standard
Discovery ST 4	1	2004	3.27	FORE + OSEM 2D	7 × 35	Gaussian 3	256 × 256	1.17 × 1.17	Loop filter: 2 mm Z-filter: standard
Discovery ST	2	2003/2004	3.27	FORE + OSEM 2D	7 × 32	Gaussian 2 or 2.57	128 × 128	2.34 × 2.34	Loop filter: 2.34 mm
Philips models	Number	Installation year	Slice thickness (mm)	Recons. method	Number of iterations (iterations × subsets)	Smoothing (mm)	Matrix	Pixel spacing (mm × mm)	Others
Gemini TF	2	2008/2010	2	LOR - RAMLA	10 × 33	Smooth B	128 × 128	2 × 2	–
Gemini GXL	1	2006	2	LOR - RAMLA	10 × NA	Smooth	128 × 128	2 × 2	–
Siemens models	Number	Installation year	Slice thickness (mm)	Recons. method	Number of iterations (iterations × subsets)	Post-reconstruction filter type and FWHM if relevant (mm)	Matrix	Pixel spacing (mm × mm)	Others
Biograph mCT 40/64	3	2009/2012	2.027	OSEM 3D	12 × 24	Gaussian 3 or 4	256 × 256	1.59 × 1.59	–
Biograph Hirez TruePoint	1	2008	3	OSEM 3D	8 × 21	Gaussian 4	336 × 336	1.02 × 1.02	–
Biograph 6 VB 20B True V	1	2006	2	FORE + OSEM 2D	8 × 21	Gaussian 4	256 × 256	1.34 × 1.34	–
Biograph 6	2	2004/2005	2	FORE + OSEM 2D	8 × 24	Gaussian 2 or 3	256 × 256	1.33 × 1.33	–
Biograph 16 VB40B	1	2004	2	FORE + OSEM 2D	8 × 24	Gaussian 3	336 × 336	1.02 × 1.02	–
Biograph LSO DUO	2	2003/2004	3.375	FORE + OSEM 2D	8 × 16	Gaussian 2	256 × 256	1.33 × 1.33	–

*NA* non applicable, *OSEM* Ordered Subset Expectation Maximization, *VPHD* VUE Point High Definition, *LOR-RAMLA* Line-of-Response Row-Action Maximum Likelihood Algorithm, *FORE* Fourier Rebinning

### Statistical analyses

The Wilcoxon signed rank test was used to compare routine and optimized values obtained from phantoms’ studies. Variances were compared with a Pitman test [10]. A significance threshold of 0.05 was adopted for all statistical analyses. Statistical analyses were performed for all centres and also for a subset of 18 centres where PSF modelling reconstruction was not available in order to assess the impact of PSF modelling on the final optimization.

## Results

All tomographs were 3D PET/CT, installed between 2003 and 2012, and consisted of 9 GE, 3 Philips, and 10 Siemens systems, with a total of 13 different models of scanners (Table 1).

In four centres (nos. 8, 13, 19, and 22), an error greater than 10 % was found for cross-calibration measurements and was later corrected by the centre’s physicist.

Four other scanners (nos. 10, 11, 14, and 20) presented a mismatch of more than 3 mm between CT and PET images, which required fixing by servicing the scanners.

### Jaszczak phantom results

**Fig. 3:**
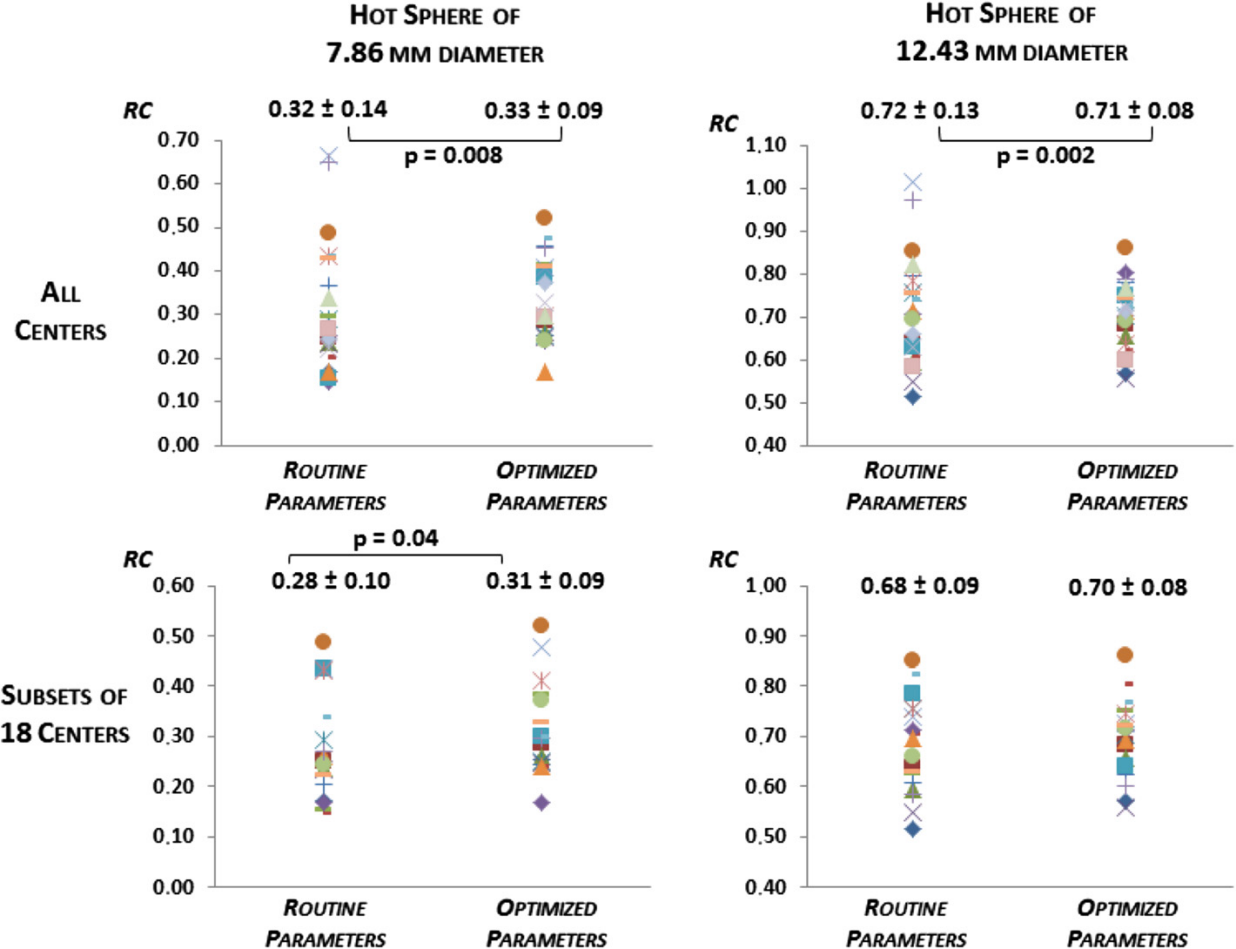
Jaszczak phantom results. Recovery coefficients (RC) obtained with routine and optimized acquisition and reconstruction parameters for the two smallest hot spheres, for all centres (*upper row*) and for the subset of 18 centres without PSF modelling (*lower row*). *P* values represent the significant test results either for comparison of means (Wilcoxon test) or for comparison of standard deviations (Pitman test)

**Fig. 4:**
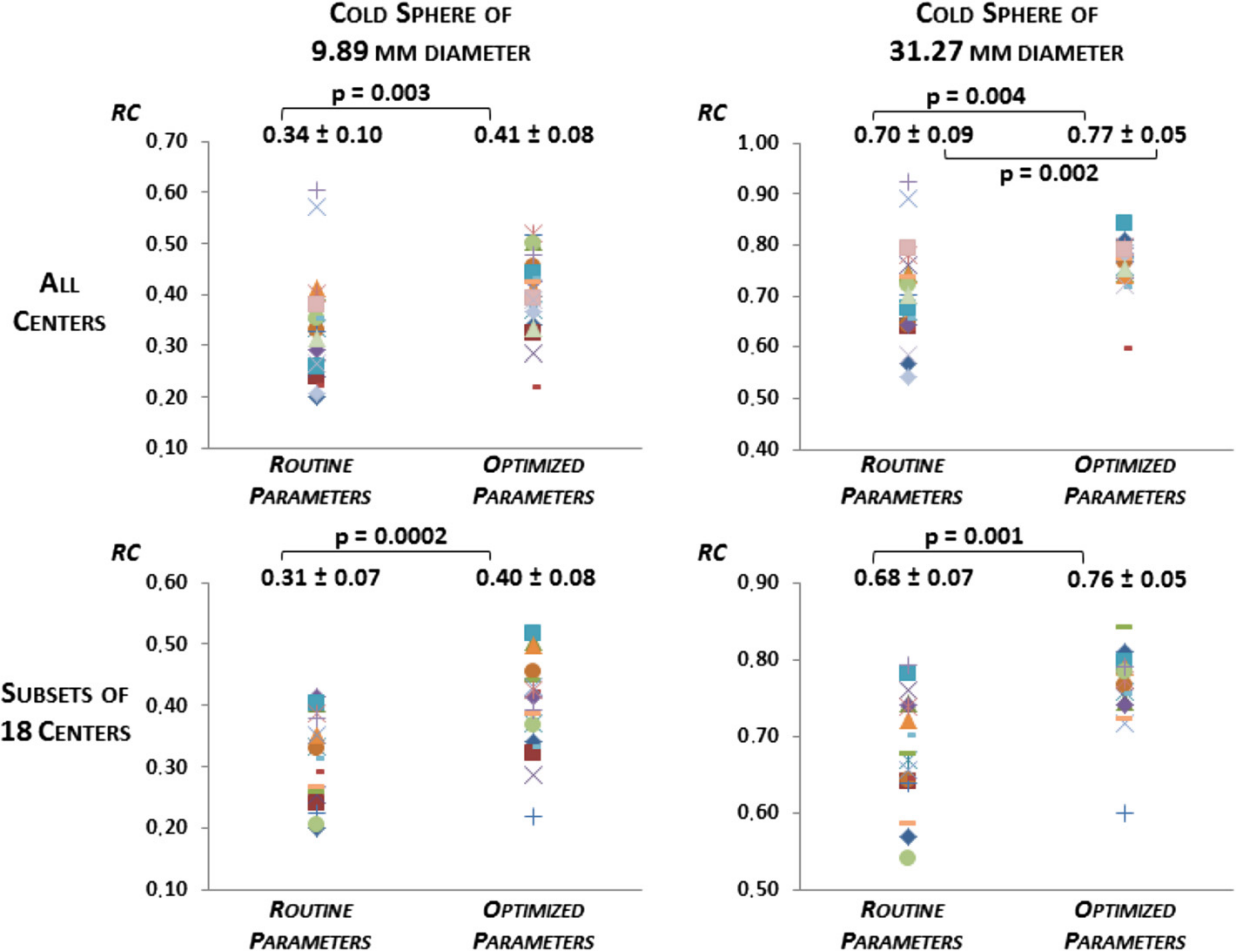
Jaszczak phantom results. Recovery coefficients (RC) obtained with routine and optimized acquisition and reconstruction parameters for the two cold spheres, for all centres (*upper row*) and for the subset of 18 centres without PSF modelling (*lower row*). *P* values represent the significant test results either for comparison of means (Wilcoxon test) or for comparison of standard deviations (Pitman test)

For all centres, the optimized RC of the two smallest hot spheres ranged from 0.17 to 0.52 (mean ± SD 0.33 ± 0.09) and from 0.56 to 0.86 (mean ± SD 0.71 ± 0.08), respectively (Fig. 3). Mean RCs were not significantly different for routine and optimized reconstructions, but the variance was significantly reduced (*p* = 0.008 and *p* = 0.002, respectively). The optimized RC of the two cold spheres ranged from 0.22 to 0.52 (mean ± SD 0.41 ± 0.08) for the smallest and from 0.60 to 0.84 for the largest (mean ± SD 0.77 ± 0.05). Mean RC significantly increased with optimized reconstructions (*p* = 0.003 and *p* = 0.004, respectively) (Fig. 4). Variance was also significantly reduced for the largest sphere (*p* = 0.002). All RC values obtained with routine and optimized parameters are presented in Table 2, with the exception of four missing data from one centre, because of an operating error.

**Table 2:** Recovery coefficient values for both reconstructions and all centres obtained from Jaszczak acquisitions

Centre number	7.86 mm Hot sphere		12.43 mm Hot sphere		14.43 mm Hot sphere		24.82 mm Hot sphere		9.89 mm Cold sphere		24.82 mm Cold sphere	
	*RP*	*OP*	*RP*	*OP*	*RP*	*OP*	*RP*	*OP*	*RP*	*OP*	*RP*	*OP*
1	0.17	0.25	0.52	0.57	0.56	0.59	0.74	0.74	0.20	0.34	0.57	0.81
2	0.25	0.29	0.64	0.68	0.76	0.77	0.95	0.95	0.24	0.32	0.64	0.79
3	0.24	0.26	0.59	0.66	0.70	0.75	0.91	0.92	0.40	0.50	0.74	0.74
4	0.23	0.25	0.55	0.56	0.74	0.73	0.93	0.91	0.25	0.29	0.76	0.75
5	0.29	0.25	0.76	0.70	0.78	0.74	0.94	0.92	0.33	0.37	0.67	0.76
6	0.49	0.52	0.85	0.86	0.90	0.91	1.01	1.01	0.33	0.46	0.64	0.77
7	0.37	0.46	0.79	0.78	0.89	0.82	1.04	0.97	0.33	0.52	0.70	0.78
8	0.20	0.24	0.61	0.62	0.70	0.70	0.92	0.91	0.22	0.22	0.64	0.60
9	0.30	0.41	NA	NA	0.87	0.82	1.06	0.99	NA	NA	0.73	0.80
10	0.15	0.24	0.71	0.80	0.80	0.85	0.99	0.99	0.29	0.42	0.64	0.78
11	0.15	0.38	0.63	0.75	0.68	0.78	0.92	0.95	0.26	0.44	0.68	0.84
12	0.17	0.17	0.71	0.71	0.73	0.73	0.91	0.91	0.41	0.41	0.74	0.74
13	0.67	0.41	1.02	0.77	0.97	0.79	1.03	0.94	0.57	0.41	0.89	0.78
14	0.44	0.30	0.78	0.64	0.72	0.70	0.93	0.94	0.40	0.52	0.78	0.80
15	0.26	0.24	0.70	0.69	0.75	0.73	0.93	0.92	0.35	0.50	0.72	0.79
16	0.65	0.45	0.97	0.79	0.95	0.84	0.98	0.98	0.60	0.48	0.92	0.79
17	0.43	0.48	0.74	0.73	0.78	0.77	0.95	0.94	0.35	0.43	0.66	0.72
18	0.43	0.41	0.76	0.74	0.78	0.76	0.87	0.86	0.39	0.43	0.74	0.77
19	0.25	0.37	0.66	0.72	0.68	0.73	0.92	0.93	0.21	0.37	0.54	0.78
20	0.27	0.30	0.58	0.60	0.75	0.76	0.97	0.97	0.38	0.39	0.79	0.79
21	0.34	0.30	0.82	0.77	0.84	0.80	1.00	0.99	0.31	0.33	0.70	0.76
22	0.22	0.33	0.63	0.72	0.76	0.82	0.96	0.97	0.27	0.39	0.59	0.72
Mean	0.32	0.33^**^	0.72	0.71	0.78	0.77	0.95	0.94	0.34	0.41^*^,^**^	0.70	0.77^*^,^**^
S.D.	0.14	0.09^***^	0.13	0.08^***^	0.09	0.06^***^	0.07	0.06	0.10	0.08	0.09	0.05^***^

*RP* routine parameters, *OP* optimized parameters

^*^Wilcoxon signed rank test, *p* < 0.05 for 22 centres; ^**^Wilcoxon signed rank test, *p* < 0.05 for 18 centres; ^***^Pitman test, *p* < 0.05

For the 18 centres where PSF modelling reconstruction was not available, the optimized RC of the two smallest hot spheres ranged from 0.17 to 0.52 (mean ± SD 0.31 ± 0.09) and from 0.56 to 0.86 (mean ± SD 0.70 ± 0.08), respectively (Fig. 3). Mean RC of the smallest hot sphere was significantly higher for optimized reconstructions *p* = 0.04), while the dispersion of values remained equivalent for both spheres. The optimized RC of the two cold spheres ranged from 0.22 to 0.52 (mean ± SD 0.40 ± 0.08) for the smallest and from 0.60 to 0.84 for the largest (mean ± SD 0.76 ± 0.05) (Fig. 4). Mean RC significantly increased with optimized reconstructions (*p* = 0.00002 and *p* = 0.001, respectively), but no significant difference was found for variances.

No significant difference could be detected (*p* = 0.61) between the values of the ratio “measured background/actual background” for routine reconstruction parameters (0.97 ± 0.13) and optimized reconstruction parameters (0.99 ± 0.08). In all centres, the SNR decreased between routine and optimized parameters, from 14.2 ± 6.16 to 9.74 ± 2.41 (mean ± SD). In the subset of 18 centres, it also decreased from 12.67 ± 4.52 to 9.70 ± 2.5 (mean ± SD).

### Image spatial resolution measurements

For all centres, the ISR ranged from 5.9 to 7.7 mm (mean ± SD 6.76 ± 0.6) with optimized parameters and from 5.5 to 7.7 mm (mean ± SD 6.98 ± 0.6) with routine parameters and did not differ significantly.

### 3D Hoffman phantom results

**Fig. 5:**
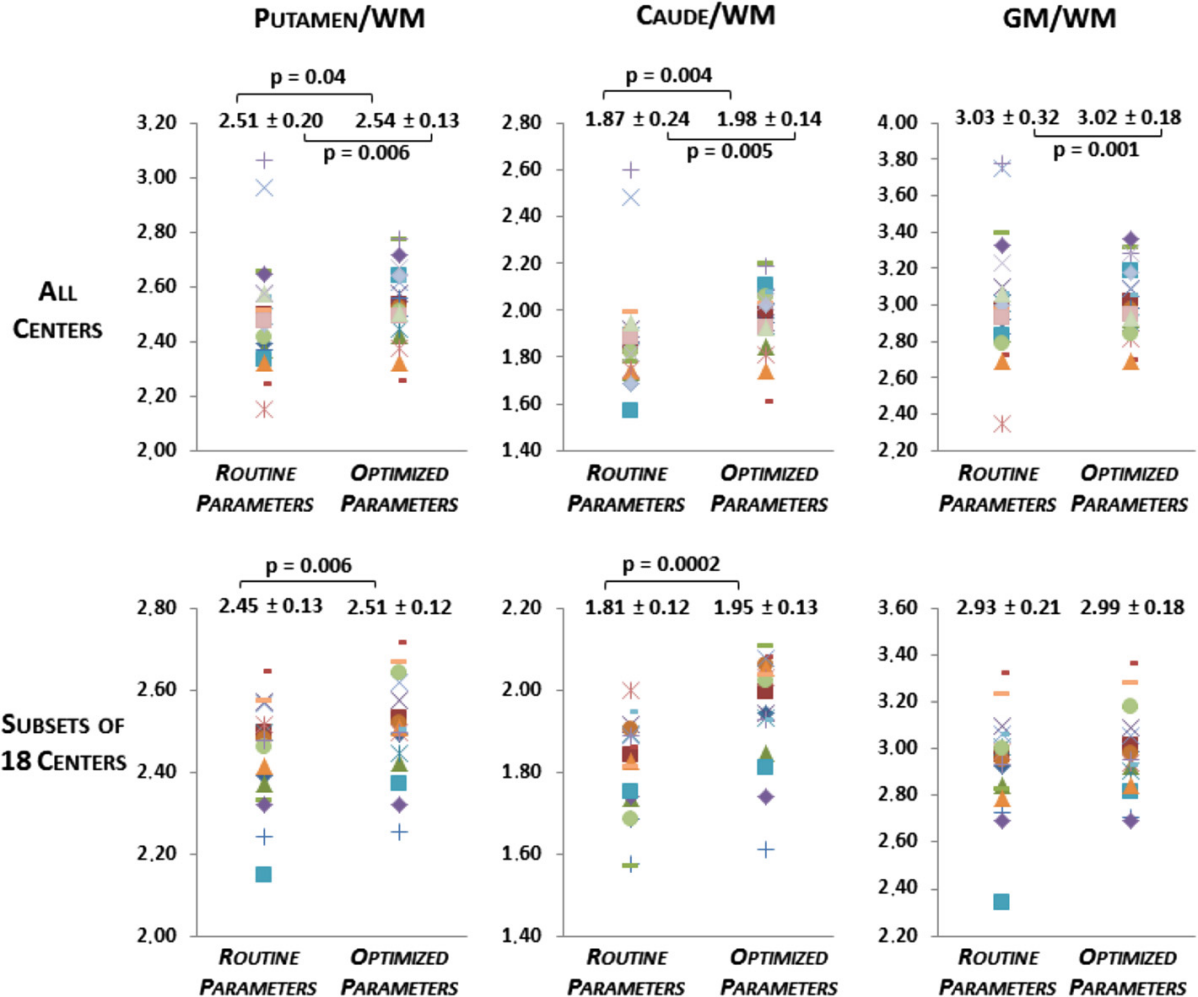
3D Hoffman phantom results. Ratio values obtained with routine and optimized acquisition and reconstruction parameters in all centres. *GM* grey matter, *WM* white matter. *P* values represent the significant test results either for comparison of means (Wilcoxon test) or for comparison of standard deviations (Pitman test)

For all centres, a significant increase of the mean C/WM (*p* = 0.005) and P/WM (*p* = 0.04) ratios was observed between routine and optimized reconstructions, but not for the other ratios. The variance was significantly reduced for the GM/WM, C/WM, and P/WM ratios (*p* = 0.001; *p* = 0.005; *p* = 0.006, respectively) (Fig. 5). In the subset of 18 centres, a significant increase of both mean C/WM (*p* = 0.0002) and P/WM ratios (*p* = 0.006) was observed. Variances were not significantly different. The ratio values for both reconstructions and all centres are presented in Table 3.

**Table 3:** Ratio values for both reconstructions and all centres obtained from Hoffman-3D acquisitions

Centre number	Right/left ratio		Anterior/posterior ratio		Putamen/WM ratio		Caudate/WM ratio		GM/WM ratio	
	*RP*	*OP*	*RP*	*OP*	*RP*	*OP*	*RP*	*OP*	*RP*	*OP*
1	1.01	1.00	1.00	1.01	2.39	2.49	1.68	1.95	2.92	2.98
2	1.00	1.00	1.01	1.01	2.50	2.53	1.84	2.00	2.98	3.02
3	1.01	1.01	1.00	1.00	2.37	2.42	1.73	1.85	2.84	2.92
4	1.01	1.01	1.01	1.01	2.57	2.57	1.92	1.94	3.09	3.09
5	0.99	0.99	1.02	1.02	2.50	2.45	1.89	1.93	3.01	2.91
6	1.02	1.02	1.03	1.03	2.48	2.52	1.91	2.06	2.96	2.98
7	1.00	1.00	1.05	1.01	2.37	2.56	1.71	2.03	2.85	2.98
8	1.02	1.02	1.04	1.05	2.24	2.25	1.57	1.61	2.73	2.70
9	1.01	1.01	1.01	1.01	2.66	2.77	1.78	2.20	3.40	3.32
10	1.03	1.03	1.00	1.00	2.65	2.72	1.86	2.08	3.33	3.37
11	1.03	1.03	0.98	0.97	2.33	2.64	1.57	2.11	2.83	3.18
12	1.01	1.01	1.03	1.03	2.32	2.32	1.74	1.74	2.69	2.69
13	1.00	1.00	1.05	1.04	2.96	2.62	2.48	2.05	3.75	3.09
14	1.01	1.00	1.05	0.99	2.15	2.37	1.75	1.81	2.34	2.82
15	1.00	1.00	1.01	1.01	2.42	2.51	1.83	2.05	2.79	2.84
16	0.99	1.00	1.02	1.03	3.06	2.78	2.60	2.19	3.77	3.28
17	1.00	1.00	1.01	1.02	2.57	2.62	1.89	2.08	3.06	3.06
18	1.00	1.00	1.05	1.05	2.52	2.50	2.00	2.03	2.99	2.94
19	0.99	0.99	1.01	1.02	2.46	2.64	1.68	2.02	3.00	3.18
20	1.00	1.00	1.01	1.01	2.48	2.50	1.89	1.93	2.93	2.95
21	1.00	1.00	1.02	1.02	2.58	2.51	1.95	1.93	3.06	2.93
22	1.03	1.02	1.00	1.01	2.57	2.67	1.82	2.04	3.23	3.28
Mean	1.01	1.01	1.02	1.02	2.51	2.54^*^,^**^	1.87	1.98^*^,^**^	3.03	3.02
S.D.	0.01	0.01	0.02	0.02	0.20	0.13^***^	0.24	0.14^***^	0.32	0.18^***^

*RP* routine parameters, *OP* optimized parameters, *WM* white matter, *GM* grey matter

^*^Wilcoxon signed rank test, *p* < 0.05 for all centres; ^**^Wilcoxon signed rank test, *p* < 0.05 for 18 centres; ^***^Pitman test, *p* < 0.05

## Discussion

Multicentre clinical trials involving imaging require procedures for image acquisition and reconstruction to be optimized to account for inter-subject profile variability and to ensure the robustness of the analysis after pooling the data and for patient follow-up. This phantom study proposed and validated a strategy for optimizing PET scanners for multicentre cerebral studies while reducing, but not minimizing, variability across centres. It involved 22 PET centres with 13 different PET/CT scanner models, most of which were of recent generation. We ensured the reproducibility of phantom measurements by sending the same technologists to the PET centres for scanner set-up.

We checked the cross-calibration between the tomograph and the dose calibration device and found a difference of more than 10 % for four centres, which was immediately corrected. The alignment between the CT and PET scanners was also systematically checked and a correction was performed if necessary. Such a correction was needed in four additional centres.

### Choice of reconstruction parameters

Harmonization across scanners and centres for multicentre cerebral imaging trials was one of the achievements of a previous study by the ADNI [11]. For that study, which included 50 centres and 17 different PET scanners, the PET centres were asked to acquire two 3D-Hoffman studies with recommended parameters. The ADNI quality-check team then checked the phantom images. For the analysis of the pooled images, a post-reconstruction smoothing filter, determined from phantom measurements, was applied to the images. This filter aimed at homogenizing the spatial resolution of the images across centres, and its application translated to a degradation of the resolution to the lowest one encountered [1].

In the present study, we chose to optimize the reconstruction parameters (with a product iterations × subsets superior to 50) and the post-reconstruction filter so that the recovery coefficients in the small cold and hot spheres would reach an optimized mean value and present limited dispersion around this optimal value. To this end, we reconstructed the images using a conventional 3D algorithm with a description of the statistics of the recorded data only, although PSF modelling reconstructions were available on the scanners that were of the more recent generations.

As expected, the reconstructions with PSF modelling provided recovery coefficients closer to 1 in the two smallest hot and cold spheres than the reconstructions without resolution modelling. However, Gibbs artefacts [12] were detected on the images at the edges of spherical objects. We therefore discarded them to avoid these artefacts and also to limit the discrepancies in RC in images reconstructed with and without resolution modelling across centres. On the other hand, we allowed algorithms with time-of-flight (TOF) modelling in two centres. It is known that state-of-the-art TOF values have an important effect on signal-to-noise ratio of whole body imaging, but that effect is negligible on smaller objects such as brain structures [13].

Conversely, in images where both spatial resolution and RC were too low, we chose to use more iterations of the algorithm in order to enhance the spatial resolution of the images and to apply a Gaussian (FWHM between 2 and 4 mm) post-reconstruction smoothing filter to the images. The pixel spacing was between 1 and 3 mm in all optimized images.

### Improving contrast recovery and dispersion of RC values

With optimized parameters, the RC significantly improved for the cold spheres, but not for the hot spheres, of close diameter. That difference between cold and hot spheres is partly related to the presence of the sphere walls, which are intrinsically cold. These walls affect the quantification to a greater extent in hot spheres than in cold spheres. Such a cold wall is specific to the phantom. One should also note that the optimized RC was higher in the hot spheres than in the cold spheres of similar diameter. The quantification in cold objects is complex, depending not only on spatial resolution but also on scatter correction and spatial sampling [14, 15], and of the non-negativity constraint of the statistical reconstruction algorithm MLEM without a specific description [16].

We also significantly reduced the variability of RC in four of the six spheres of the Jaszczak phantom. As shown in Figs. 3 and 4, this reduction of variability was mostly due to the suppression of outliers by discarding PSF modelling reconstruction algorithms.

It should be emphasized that the RC measurements were obtained with in-house software, which was developed with great care to ensure reproducibility and precision. In particular, the VOIs were not drawn directly on the PET images but were automatically segmented on CT images and optimized with knowledge from the phantom specifications.

### Improving quantification and variability in small brain structures and image uniformity

We checked the quantification in the striatum of the Hoffman brain phantom and observed an increase in activity concentration measured in the putamen and caudate, when compared to non-optimized images. We also significantly reduced after the optimization the dispersion of values for putamen, caudate, and grey-matter-to-white-matter ratios.

Finally, the good uniformity of the images across the field of view was confirmed by the ratio of right-to-left activity concentration and anterior-to-posterior activity concentration measured on the Hoffman brain images. Additionally, as expected, the choice of the reconstruction mode parameters had no impact on the ratio of activity concentration in the right-to-left and frontal-to-occipital regions of interest. Those ratios should not be affected by the spatial resolution. However, they are likely affected by low frequency variation resulting from scatter, uniformity, and attenuation corrections. Attempts to take into account these effects before pooling data sets from multiple centres were suggested by Joshi et al. [1], but the results were not considered as convincing by the authors. In the present work, the compensation for scatter, random coincidences, and attenuation was performed using the manufacturer’s latest techniques, with slight differences in the implementation for the different scanner models. The Hoffman phantom also enabled us to verify that the quantification in typical small structures of the brain, such as the caudate, was improved after the reconstruction optimization.

### Image spatial resolution and signal-to-noise ratio

On average, image spatial resolution was slightly improved, despite a marked decrease for the centres equipped with reconstruction algorithms including TOF and PSF modelling. This improvement is likely due to the increase in the number of iterations in the other centres. As expected, this improvement is counterbalanced by a decrease of the signal-to-noise ratio. We checked with Hoffman phantom acquisitions that this reduction did not impact the visual aspect of the images.

### Variability across centres

The variability across centres was reduced but not minimized by this work. For that, it would have been necessary to apply post-filtering tailored per site according to ISR. We deliberately chose not to do this in order to preserve the spatial resolution. This latter goal was achieved for the 18 centres without PSF modelling reconstruction.

In order to account for the residual variability across centres, we are currently designing a statistical model that takes into account the centre effect (as assessed by the phantom studies). Several other parameters have been shown to influence the variability of the measurements across centres, including injected dose per kilogram, delay between the injection and the scan acquisition, patient positioning, weight, and glycaemia, and these have been extensively discussed by Boellard [17, 18]. We will add to the statistical model these additional acquisition and patient-related parameters (as assessed by an ancillary monocentric study involving 300 subjects), and we will test it on the MEMENTO cohort.

If the statistical model happens not to be sufficiently effective, our approach could be updated in a second phase according to the strategy proposed by Lasnon et al. [19]. The latest advances in reconstruction schemes could be used to reconstruct images together with an adequate post-reconstruction filtering, offering the best compromise in image quantification for multicentre studies.

## Conclusions

This work was undertaken by CATI’s team in charge of harmonization, quality check, and analysis of multicentre brain PET or SPECT acquisitions. The proposed procedure for PET imaging optimization enabled the production of images with a more homogenous spatial resolution across the centres. The spatial resolution was also preserved thanks to the use of statistical image reconstructions with iterations × subsets of at least 50, a Gaussian smoothing post-reconstruction filter with a FWHM between 2 and 4 mm, and a pixel spacing between 1 and 3 mm. The uniformity across the fields of view of the scanners was good.

We provided recommendations to each centre for minimizing the influence of other factors such as injected dose and interval post-injection. In addition, centres were qualified after the analysis of the images of a first test patient. CATI has now received more than 3000 PET images.

This step of optimization of the image characteristics acquired at different centres will thus allow us to account for the residual variability, which could be handled in the final statistical analysis using adequate modelling.

### Ethical approval

This article does not contain any studies with human participants or with animals performed by any of the authors.
